# Neurostimulation in the Middle East: What Do We Know So Far? A Narrative Review

**DOI:** 10.3390/brainsci15101033

**Published:** 2025-09-24

**Authors:** Ahmad H. Almadani, Sumaiya Nishat, Ghada K. Alrashed, Abdullah J. Alghanim, Ayedh H. Alghamdi, Mohammed A. Aljaffer

**Affiliations:** 1 Department of Psychiatry, College of Medicine, King Saud University, Riyadh 11451, Saudi Arabia; aayedh@ksu.edu.sa (A.H.A.); maljaffer@ksu.edu.sa (M.A.A.); 2 Department of Psychiatry, King Saud University Medical City, King Saud University, Riyadh 11362, Saudi Arabia; 3 Department of Special Education, Alamad International School, Riyadh 14227, Saudi Arabia; 4 Department of Psychiatry, King Fahad Hospital of The University, Khobar 34445, Saudi Arabia

**Keywords:** electroconvulsive therapy, Middle East, neurostimulation, transcranial magnetic stimulation

## Abstract

Mental health disorders are increasingly being recognized as a major global challenge. In the Arabic-speaking Middle East and North Africa (MENA) region, this challenge is compounded by sociocultural stigma, political instability, and limited mental health infrastructure, all of which restrict access to effective care. While neurostimulation modalities such as electroconvulsive therapy (ECT) and repetitive transcranial magnetic stimulation (rTMS) have proven effective and are gaining traction, their use in the MENA region remains limited and underexplored. This narrative review aims to bridge critical gaps by examining knowledge levels, attitudes, perceptions, and the clinical application and accessibility of ECT and rTMS across Arabic-speaking countries. We searched multiple databases using keywords related to neurostimulation and psychiatry, covering all 22 Arabic-speaking MENA countries. Studies were included if they were published in English and were related to psychiatric applications of ECT or rTMS. Findings were categorized by geography and grouped into four thematic domains: knowledge, perception, availability, and clinical use. The findings revealed an uneven distribution of neurostimulation research and services across the region; ECT is more established than rTMS. Additionally, public awareness remains low, and high levels of stigma persist. Among clinicians, psychiatrists tend to support neurostimulation, while general medical staff show mixed opinions. rTMS is gaining clinical interest but remains limited in accessibility due to high costs and limited infrastructure. Although neurostimulation should be more widely implemented in psychiatry in the MENA region, it is still underrecognized and underused. Region-specific strategies addressing stigma, training gaps, and policy standardization are essential to optimize neurostimulation use and its public acceptance.

## 1. Introduction

Mental health is a critical component of overall well-being, with mental disorders ranking among the leading causes of disability worldwide [1]. The World Health Organization (WHO) defines mental disorders as clinically significant disturbances in cognition, emotion, or behavior, often resulting in impaired functioning and diminished quality of life. Furthermore, a WHO report indicated that in 2019, approximately one in eight individuals globally experienced a mental health condition, with depression and anxiety being the most prevalent [2]. The COVID-19 pandemic further intensified this burden, triggering a 25% increase in global rates of anxiety and depression [2]. These figures highlight an urgent need for effective, accessible, and scalable treatment strategies for psychiatric illnesses. While pharmacotherapy and psychotherapy remain foundational in treating mental disorders, a significant number of individuals fail to respond to conventional approaches, known as treatment-resistant cases [3]. Such treatment-resistant cases have accelerated the exploration of neurostimulation as an alternative treatment option in the field of psychiatry.

Neurostimulation refers to a range of techniques that apply electrical or magnetic stimulation to modulate neural activity [4]. These methods fall into two broad categories: invasive (such as deep brain stimulation and vagus nerve stimulation) and non-invasive approaches, such as electroconvulsive therapy (ECT), repetitive transcranial magnetic stimulation (rTMS), and transcranial direct current stimulation (tDCS). Among the non-invasive options, ECT and rTMS have gained considerable clinical traction in psychiatry.

ECT is one of the oldest neurostimulation techniques used in psychiatric practice. It induces controlled seizures under anesthesia and remains highly effective for severe and treatment-resistant conditions such as major depressive disorder, bipolar disorders, catatonia, and schizophrenia [5]. Furthermore, it is considered safe by major psychiatric bodies and practitioners [6]. For instance, the U.S. Food and Drug Administration’s 2018 reclassification of ECT devices from Class III to Class II for certain indications may affect the application of this therapy, as it facilitates the continued availability of ECT devices worldwide and helps decrease the stigma associated with this procedure by acknowledging its safety and effectiveness [7]. In contrast, rTMS employs magnetic pulses to target specific brain regions and was first demonstrated to non-invasively stimulate the human motor cortex in 1985 [8]. It does not require anesthesia and is generally well tolerated [9]. Treatment outcomes for rTMS depend on factors such as frequency, intensity, and duration of stimulation [10]. While initially approved for treatment-resistant depression, rTMS is being explored for a broader range of psychiatric conditions, including obsessive–compulsive disorder, post-traumatic stress disorder, schizophrenia, and substance use disorders [11].

The Middle East and North Africa (MENA) region, encompassing 22 Arabic-speaking countries, spans a diverse geographical and sociopolitical landscape. It includes several subregions: North Africa, which comprises Algeria, Egypt, Libya, Morocco, and Tunisia; the Levant, including Iraq, Jordan, Lebanon, Syria, and Palestine; the Arabian Peninsula, consisting of Bahrain, Kuwait, Oman, Qatar, Saudi Arabia, the United Arab Emirates (UAE), and Yemen; and the Horn of Africa and Indian Ocean islands, which include Somalia, Sudan, Djibouti, and Comoros. Although unified via language (Arabic), these countries differ greatly in their economic conditions and political systems. Mental disorders impose a considerable burden across MENA, with the global burden of disease showing elevated disability-adjusted life years (DALYs) related to psychiatric conditions [12]. Contributing factors include ongoing political conflicts, forced displacement, and fragile healthcare systems [13]. Mental illnesses in such regions are significant, with depression and anxiety being the most commonly reported, disproportionately affecting women, refugees, low-income individuals, and those with chronic diseases [14].

Despite a growing awareness of mental health concerns, stigma continues to hinder access to care [15]. Negative cultural perceptions, gender-based disparities, and reliance on traditional healing practices often prevent timely diagnosis and treatment [16]. Nevertheless, the past decade has witnessed increased efforts in research and policy discussions aimed at improving mental health services [15,17]. However, research on advanced psychiatric interventions, particularly neurostimulation, remains scarce in this region. This narrative review seeks to address this gap by examining the various aspects related to ECT and rTMS across Arabic-speaking MENA countries in an effort to contribute to the evolving mental health landscape in the Arab world. More specifically, this review seeks to analyze Ref. [1] knowledge levels; Ref. [2] perceptions, attitudes, and opinions; Ref. [3] uses; and Ref. [4] access and availability.

## 2. Methods

A comprehensive literature search was conducted across various databases, including PubMed, Scopus, Web of Science, EBSCO and MEDLINE, ProQuest, and Google Scholar. Additional databases such as DOAJ, JSTOR, and ScienceDirect were also explored but excluded due to the repetitiveness of the findings and a lack of relevant results. The following keywords were used in combination during the search process: “Neurostimulation,” “Neuromodulation,” “Electroconvulsive Therapy,” “Transcranial Magnetic Stimulation,” “Repetitive Transcranial Magnetic Stimulation,” “Psychiatry,” and “Mental Disorders,” along with the names of all 22 Arabic-speaking MENA countries. There were no restrictions on the publication date, but only studies published in English and directly related to psychiatric applications of ECT and rTMS were included. Case reports, non-peer-reviewed publications, and studies not addressing psychiatric applications of neurostimulation were excluded. After applying the inclusion and exclusion criteria, a total of 46 papers were included in this review. Zotero reference management software version 7.0.21 was used to organize and manage the selected literature throughout the review process [18]. For organizational purposes, the 22 Arabic-speaking countries were categorized geographically into four subregions: (1) the Arabian Peninsula, (2) the Levant, (3) North Africa, and (4) the Horn of Africa and Indian Ocean Island states. In addition to the papers selected, other international studies relevant to the scope of the review were included to address specific gaps in the topic.

## 3. Knowledge Levels

The knowledge and implementation of neurostimulation techniques such as ECT and rTMS vary widely across the Arab world, as evidenced by studies from Saudi Arabia and Iraq [19,20]. These differences are influenced by a complex interplay of economic development, sociocultural attitudes, and healthcare infrastructure [21].

In the Arabian Peninsula, the Gulf Cooperation Council (GCC) states, despite being affluent, still face challenges in mental health services, partially due to persistent stigma and strong reliance on traditional belief systems [15]. Of all the countries in this region, Saudi Arabia has produced the most research into ECT and rTMS, yet public awareness remains low. A study conducted in the city of Hai’l in Saudi Arabia concluded that only 3.7% of surveyed individuals recognized ECT as a safe treatment for depression, and 97.3% were unaware of TMS [15]. In another study, a positive correlation was found between educational attainment (e.g., medical students, residents, and practicing psychiatrists) and neurostimulation-related awareness, highlighting the role of formal psychiatric education in shaping attitudes and knowledge [19]. Institutional gaps also remain. A national audit in Oman reported the absence of standardized policies and clinical guidelines for ECT administration [22]. In Bahrain, ECT is recognized as a therapeutic option for treatment-resistant depression (TRD), particularly after multiple medication and psychotherapy failures [23]. Notably, a retrospective study revealed that more than half of the individuals who received ECT in Bahrain between 1984 and 1987 had initially sought psychiatric care at the Bahrain Psychiatric Hospital as self-referrals [24]. Awareness of rTMS in the GCC remains limited, given its relatively recent clinical introduction.

In North Africa, Egypt is the regional leader in both research output and the clinical use of neurostimulation, with formal ECT training integrated into psychiatric education [25]. While public knowledge is moderate, stigma and misconceptions remain persistent [26,27]. Furthermore, it was observed that other North African countries, such as Algeria, Libya, and Tunisia, have limited research on this topic, while Mauritania has no published studies. In Libya, efforts have been initiated to improve access to mental healthcare, supported by the WHO [28]. Similarly, articles on rTMS produced outside of Egypt were found to be scarce [29].

Most Arabic-speaking countries of the Levant region face challenges due to economic constraints and other factors. Their healthcare systems, including mental health services, have been constrained by limited resources. Public knowledge is low, and misconceptions remain persistent. For example, in Jordan, the accuracy rate on questions about ECT was only 45.1% [30]. Iraq has a longstanding history of ECT use [20]. Nevertheless, in 2009, as part of a national quality and standards initiative to rebuild mental health services, its Ministry of Health introduced an ECT policy aimed at formalizing practices and improving treatment consistency [31]. Searches for studies on rTMS in the Levant region yielded no results, reflecting the nascent or absent status of rTMS research and clinical implementation across Levantine Arabic-speaking countries.

Regarding the Horn of Africa and Indian Ocean Islands, Sudan produced the most results, with moderate awareness found in urban areas such as Khartoum [32]. However, unmodified ECT (i.e., without anesthesia) remains common [33], and continued professional development is limited [32]. Somalia, Djibouti, and the Comoros demonstrate extremely low mental health literacy, with no available data on neurostimulation.

While countries such as Saudi Arabia and Egypt have made notable progress, even these systems are somewhat hindered by limited public understanding and a lack of structured quality assurance frameworks. Economic disparities in some countries, sociocultural stigma, and the absence of standardized clinical policies are potential barriers. This regional overview highlights a persistent gap between the knowledge of healthcare providers and public awareness of neurostimulation techniques across the Arab world.

To bridge these gaps, region-specific interventions are needed to improve knowledge of neurostimulation. One necessary intervention is the development of educational campaigns, webinars, and lectures targeting the public and non-psychiatric mental health workers. Our recommendation in this regard aligns with the recommendation of a United States (US) and Canadian study [34] that surveyed members of the public to assess their awareness and understanding of rTMS, revealing relatively poor public perceptions of rTMS. In that study [34], the authors recommend targeted educational initiatives to enhance awareness and acceptance. Furthermore, in the United Kingdom (UK), a study involving medical students demonstrated that a one-hour technology-enhanced learning and teaching session about ECT significantly improved their knowledge and attitudes toward the treatment [35]. These findings highlight the value of educational interventions in improving perceptions of neurostimulation and emphasize the need to extend such efforts to the public and non-specialist mental health workers. A second approach is to integrate neurostimulation training into medical school curricula and residency training programs and develop subspecialized fellowship training programs in neurostimulation. This is especially important, since ECT exposure in training programs varies tremendously. For instance, a study showed that three-quarters of psychiatry residency programs in the US and Puerto Rico mandate clinical experience with ECT; however, only 57% offered a specific ECT rotation as part of the training [36]. To address such inconsistencies and support more standardized training, a randomized controlled trial in the US demonstrated that psychiatry residents who received a simulation-based ECT curriculum showed significantly greater improvements in knowledge, procedural proficiency, and confidence compared to those who received only traditional instruction [37]. These findings suggest that simulation-based training not only enhances residents’ comfort and competence with ECT but also offers a practical solution for programs with limited clinical resources, making it a strong model for wider implementation [37]. A third approach involves advocating for policy reforms that support standardized, evidence-based practices, especially given the existence of well-established clinical guidelines that outline the safe and effective use of neurostimulation therapies [6,38].

## 4. Perceptions, Attitudes, and Opinions

Among the general population, apprehension about ECT is widespread [26]. Despite the widely documented success of ECT, misconceptions such as permanent memory loss or brain damage are common. For instance, a study conducted in Saudi Arabia found that only 3.7% of participants believed that ECT could be used to treat TRD despite it being considered safe [15]. These fears are often reinforced by sensationalized portrayals in films and television, which depict ECT as a coercive or punitive act. Furthermore, mental illness itself is frequently attributed to spiritual or supernatural causes, such as the evil eye, black magic, or a lack of religious faith. In a 2021 study, 67.3% of participants believed that depression stemmed from a lack of faith, 45.5% attributed it to the evil eye or sorcery, and 56% endorsed faith healing as a valid treatment [15]. Alarmingly, 89.4% believed that depression could be overcome through sheer willpower, highlighting a fundamental misunderstanding of psychiatric disorders as medical conditions [15].

Despite these widespread misconceptions, firsthand experience with ECT, either as a patient or caregiver, often leads to more positive views. For instance, in Egypt, caregivers who initially regarded ECT as “dangerous” or “inhumane” shifted toward favorable opinions following their relative’s treatment, recognizing its clinical efficacy and safety [39]. A similar trend was observed in Sudan, where 70.7% of family members expressed support for ECT use in emergencies, and over half of the patients themselves reported a positive overall attitude toward the treatment [32]. Among healthcare providers, psychiatrists generally demonstrate supportive views toward ECT, acknowledging its value in cases of severe or treatment-resistant illnesses [40]. However, non-psychiatric medical professionals often mirror public skepticism, though to a lesser extent [41]. A study in Oman revealed that medical professionals outside psychiatry expressed reservations about ECT, reflecting gaps in inter-professional psychiatric training [22].

In Iraq, ECT remains one of the most accessible and used treatment modalities. Moreover, the shortage of newer psychiatric medications and infrastructure has led clinicians to view ECT as a vital therapeutic tool rather than a last resort [42]. Additionally, a cross-sectional study drawing comparisons between medical students in three countries, two from MENA and one from the West, showed that in the UK, 75% stated that they would receive ECT themselves, which was considerably higher than in Iraq and Egypt, at 56% and 29%, respectively [43]. In Lebanon, even among the educated population, including hospital staff and students, perceived stigma remains high, as reported by Hayek et al. in 2021 [44]. In certain countries, such as Syria and Palestine, attitudes are difficult to assess due to severe workforce and training shortages. Gender differences in perception have also emerged, with studies suggesting that men are more likely than women to accept medical interventions, whereas women often prefer traditional or spiritual treatments, a preference influenced by social roles and religious expectations [24,45].

Awareness of rTMS remains particularly low, confined mostly to psychiatric specialists [46]. Moreover, its relative novelty, limited availability, and higher cost contribute to its relatively low public profile [46]. Nonetheless, among psychiatrists, rTMS is increasingly viewed as a promising alternative for patients who are either unresponsive to ECT or hesitant to undergo seizure-based treatments [47]. A survey of psychiatrists in Saudi Arabia found that 79% held positive attitudes toward rTMS, highlighting growing clinical interest in this non-invasive modality [47].

Efforts to overcome negative attitudes and perceptions toward ECT and rTMS in general, particularly among the public and non-psychiatric healthcare workers (such as general practitioners), are warranted. One way to achieve this goal is by delivering educational campaigns targeting the aforementioned groups to fight the stigma attached to psychiatry, particularly the application of neurostimulation. This approach aligns with findings from a South African study showing that increased exposure to psychiatric conditions and personal familiarity with mental illness (personal or family experiences of mental illness) are associated with significantly lower stigma levels, underscoring the importance of targeted interventions, especially among medical students and future healthcare providers [48]. Even brief educational interventions—such as a short video—have been shown to significantly improve attitudes toward ECT among healthcare trainees, suggesting the potential effectiveness of such approaches [49]. Another important approach is to encourage various stakeholders, such as health policymakers and non-psychiatric mental health workers, to become advocates for neurostimulation. This is especially relevant given global findings showing that the use of ECT is linked to a nation’s level of financial investment in mental healthcare [50]. Collaborating with media staff to improve the perception of neurostimulation is also valuable, especially as many individuals form their understanding of treatments like ECT through popular media. For instance, in one study from the UK, both medical students and members of the general public reported that movies and television were their primary sources of information about ECT [51].

## 5. Uses

ECT is used across the Arab world to treat severe psychiatric disorders such as schizophrenia, mania, TRD, and catatonia [39,52]. One difference that can be noted between many of the Arab nations and the West is that the prevalence of ECT use is higher for disorders such as schizophrenia than for mood disorders such as TRD [22,53]. In Oman, schizophrenia was the most common diagnosis among ECT recipients (32.5%), followed closely by major depressive disorder (31.5%) [22]. Furthermore, the clinical follow-up was inconsistent, with only 66% of cases in Oman receiving post-session evaluations [22]. As for Qatar and Saudi Arabia, ECT is primarily employed for cases of TRD, especially among inpatient populations [54,55]. While the UAE, Kuwait, and Bahrain have smaller populations, these countries maintain operational neurostimulation services, although published data on their use remain limited [56,57,58]. While still in its early stages, rTMS is beginning to see clinical application in the Arabian Peninsula. Saudi Arabia has introduced rTMS in some psychiatric centers and added protocols for the management of major depressive disorders [59]. Oman conducted a pilot study involving 49 patients with refractory depression who completed a standard 20-session rTMS protocol [60]. The study reported a 37% response rate, indicating promising therapeutic potential [60]. Similarly, in the UAE, rTMS has been used for TRD, with a reported response rate of 58% among treated patients [56].

In North Africa, Egypt has the highest documented usage. For instance, Assiut University Hospital administers 15–20 ECT sessions daily for disorders such as bipolar disorder (48.3%), schizophrenia (24.9%), and depression (17.9%) [39]. Tunisia follows similar clinical patterns, though on a smaller scale, with most reported cases involving bipolar mania (47.1%), followed by schizophrenia (35.3%) and depression (4.7%) [61]. Algeria has begun using rTMS as an adjunct for TRD, particularly when medication is ineffective or contraindicated [62]. Meanwhile, Libya has experienced a steep decline in ECT use, probably due to the disruption of psychiatric services amid political instability [28].

In the Levant and the Horn of Africa, uses are sparsely documented. In Iraq, ECT is often preferred over medication due to shortages, and despite the challenges of prolonged conflict, it remains in use, particularly in tertiary centers, though anesthesia shortages continue to necessitate unmodified administration in some settings [63]. Psychiatric hospitals in Sudan’s capital, Khartoum, predominantly admit severe cases of schizophrenia, mania, and depression, with ECT being routinely used for these conditions [33]. However, the use of unmodified ECT remains widespread and systematic post-treatment evaluation is often lacking [33]. As for rTMS, no clinical usage data were identified from either the Levant or the Horn of Africa/Indian Ocean Islands. This absence reflects the absence or early stage of rTMS implementation in these regions, probably due to high costs, a lack of equipment, and limited professional training.

Several approaches may be considered to further promote the use of neurostimulation treatment modalities. Strategies that could improve application include increasing knowledge of the various indications and clinical uses, raising awareness of the robust evidence behind ECT and rTMS, especially for ECT, developing best-practice guidelines for treatment delivery, and exploring the feasibility of outpatient ECT for appropriate patients. This is particularly important given that non-clinicians, such as patients, caregivers, and the general public, most frequently cite “limited evidence of the treatment’s effectiveness” and “lack of understanding of the intervention” as the main barriers to accepting ECT and rTMS [64]. Addressing these knowledge gaps through education and evidence dissemination is therefore crucial to improving acceptance and access.

## 6. Access and Availability

Access to neurostimulation treatments across Arabic-speaking countries seems to vary significantly. These disparities are largely shaped by economic stability, healthcare budgets, workforce limitations, and the development of medical infrastructure [21,33]. While several urban centers offer both ECT and rTMS, such services are often concentrated in large cities, leaving rural and lower-income areas underserved [21,33].

In the Arabian Peninsula, ECT is generally available in tertiary psychiatric centers, though disparities persist. However, the level of access varies by country and even within regions. For example, in Saudi Arabia, services are mostly found in major urban areas, while smaller cities still face major barriers such as limited public awareness and logistical difficulties [15,65]. In the UAE, ECT is offered at specialized inpatient centers such as Al Amal Hospital, but coverage outside urban hubs remains limited [66,67]. In Oman, only two hospitals—Sultan Qaboos University Hospital and Al Masarra Hospital—currently provide ECT. Although limited, Oman has an rTMS facility, signaling early but promising infrastructure development [60].

North Africa presents a mixed picture, with some countries, such as Egypt, having developed a neurostimulation network among the urban cities in the region, while countries such as Algeria, Morocco, and Tunisia have more limited access. No results were obtained from Mauritania, another country in the North African region. Moreover, in the Levant, access to neurostimulation is more limited, with some countries such as Syria and Palestine having little to no access. ECT is routinely provided at major public institutions in Cairo and Alexandria, where equipment and trained staff are present. Several studies in Egypt have confirmed the availability of ECT in both university and government hospitals, though rural coverage remains poor [39]. Despite its presence in the guidelines of the Egyptian Psychiatric Association for the Management of Treatment-Resistant Unipolar Depression, all the available papers are from clinical trials in university hospitals, while broader availability and general practice remain uncertain [25,26,27]. In contrast, Libya has reported damaged or non-functional ECT facilities [28]. Tunisia offers ECT within psychiatric hospitals, but no information is available on rTMS availability [61].

In the Horn of Africa and Indian Ocean Islands, accessibility is generally limited. In Sudan, ECT is available in psychiatric institutions in Khartoum, though the equipment is outdated and the use of unmodified ECT remains common due to shortages of anesthesia [33]. No data were available regarding rTMS access in Sudan or surrounding countries. There is no documented availability of either ECT or rTMS in Djibouti or the Comoros.

Thus, while ECT is more widely accessible than rTMS across the Arab world, both modalities face challenges including urban–rural disparities, workforce shortages, outdated equipment, and minimal national coordination [15,47]. More substantial efforts are therefore needed to improve access and availability of ECT and rTMS, particularly in remote and rural areas where mental health services remain unevenly distributed. A systematic review of rural–urban disparities among individuals with mental health conditions in North America found that more than half of the studies reported worse outcomes for rural patients, including higher suicide rates, diagnostic challenges, and limited access to care [68]. However, a large-scale United States Department of Veterans Affairs study showed that mental health service access among rural veterans increased significantly following policy changes, including a requirement for the availability of mental health services through community-based clinics [69]. These findings support the value of developing policies that promote the equitable distribution of mental health services, including neurostimulation technologies, and coordinated efforts between urban and rural healthcare providers. Nonetheless, a Canadian study found that even when ECT is geographically accessible to most citizens, significant barriers persist—particularly the limited availability of trained professionals, treatment facilities, and adequate funding [70]. To address these challenges, it is necessary to invest in infrastructure to further increase mental health facilities and overcome logistical challenges; train healthcare professionals to be prepared to deliver ECT and rTMS; and provide the required human resources, medications, and equipment. Moreover, advocating for the inclusion of neurostimulation interventions in insurance policies could also ease the accessibility and availability of such interventions. This idea is supported by findings from a US study in which psychiatrists identified the lack of insurance coverage as a major barrier to accessing treatments such as TMS [64]. Collaboration between mental health workers, nursing personnel, and other departments, such as the anesthesia department, is also key to facilitating access and availability of ECT and rTMS.

## 7. Strengths and Limitations

This narrative review has strengths and limitations. In terms of strengths, it is one of the few articles that have attempted to discuss the topic of neurostimulation in the Middle East. It also examined multiple crucial aspects related to the topic, such as knowledge, access, and availability. Moreover, it presented a comprehensive overview of the subject in the region, identifying gaps in the various elements discussed in this paper and offering insights.

However, this narrative review also has some limitations. Unlike other study designs, such as systematic reviews, this narrative review does not follow a predefined protocol, making it somewhat difficult to replicate the review process. The narrative review design also introduces selection bias into the included studies, which is another drawback. In particular, restricting our literature review to English-language publications may also be viewed as a bias. In addition, in an effort to be inclusive, we restricted our search keywords to general phrases such as “Mental disorders” rather than specifically searching for specific mental disorders such as depressive, bipolar, and psychotic disorders. As such, a more rigorous research methodology, such as conducting systematic reviews and meta-analyses of the topic, could yield more robust results. Furthermore, while attempting to be comprehensive and inclusive, the review omitted tDCS in favor of focusing on the two most well-established neurostimulation therapies in the field of psychiatry: ECT and rTMS. In contrast, tDCS, while promising and superior to sham in meta-analyses, has small effect sizes and lacks the data maturity of ECT and rTMS [71]. Nonetheless, tDCS has practical advantages such as low cost, portability, ease of use, and the potential for home-based therapy, which could increase access in the MENA region [72].

## 8. Conclusions

Despite the potential of ECT and rTMS to treat psychiatric disorders throughout the MENA region, factors such as stigma, unequal access, and a lack of professional training appear to limit their use [15,30,41]. These factors and challenges are summarized in Table 1. Thus, addressing these challenges requires the development of interventions and policies focused on mental health. Moreover, it is important to remain sensitive to cultural differences. Increased funding for neuromodulation infrastructure is crucial. Furthermore, more medical schools should include ECT and rTMS education in their curricula. Likewise, neurostimulation should also be incorporated into psychiatry residency training programs. Additionally, specialized fellowship programs in neurostimulation are also needed, as well as recognizing it as a subspecialty in the psychiatric field. It is also necessary to engage various stakeholders to improve the availability of and access to ECT and rTMS. Such stakeholders include health policymakers, regulatory bodies, funding agencies, insurance companies, and non-mental healthcare workers. Moreover, more local, national, and international collaboration, clinical- and research-wise, is also warranted.

## Figures and Tables

**Table 1:** Summary of ECT and rTMS status in the Middle East.

Subregion	Culture and Attitude	Infrastructure and Access	Knowledge and Clinical Use
The Arabian Peninsula (e.g., Saudi Arabia, UAE, and Oman)	Strong influence of traditional beliefs and persistent stigma, even in affluent nations [15]. Public apprehension is high; mental illness is often attributed to other causes, such as supernatural causes [15]. Psychiatrists are generally supportive, but other medical professionals show reservations [40,41,47].	ECT is generally available in tertiary psychiatric centers but concentrated in major urban areas, leading to rural disparities [65,66,67]. rTMS is being introduced in some centers but is not widely available [56,59,60].	ECT is used for TRD and schizophrenia [22,54,55]. rTMS is emerging for TRD [56,59,60].
The Levant (e.g., Iraq, Jordan, and Lebanon)	High levels of perceived stigma, even among educated populations and hospital staff [44]. In conflict-affected areas such as Iraq, clinicians view ECT as a vital tool due to medication shortages, not a last resort [42].	Access to ECT is severely limited by economic constraints and political instability. Services are sparse, with little to no access in countries like Syria and Palestine. Anesthesia shortages in Iraq can lead to the use of unmodified ECT [63]. No documented rTMS availability.	Public knowledge about ECT is low, with persistent misconceptions [30]. Iraq has a long history of ECT use and has established a national policy to formalize its practice [20,31].
North Africa (e.g., Egypt, Tunisia, and Libya)	Stigma and misconceptions persist, though firsthand experience often leads to more positive views from patients and caregivers (e.g., Egypt) [26,27,39].	A mixed picture: Egypt has a relatively developed network in urban centers, while access in other nations is more limited [39]. Political instability has led to damaged or non-functional facilities in some areas (e.g., Libya) [28]. Rural coverage is generally poor, even in countries with established services [39].	Egypt is the regional leader in research, clinical use, and formal ECT training in medical education [25]. High documented use of ECT in Egypt for bipolar disorder, schizophrenia, and depression [39,61]. rTMS use is emerging in some countries (e.g., Algeria) but is not widespread [29,62].
Horn of Africa and Indian Ocean Islands (e.g., Sudan and Somalia)	Positive attitudes are often reported by patients and families with direct experience with ECT (e.g., Sudan) [32].	Access is generally very limited or non-existent [33]. In Sudan, ECT is available in the capital city, but equipment is often outdated [33]. Frequent use of unmodified ECT due to shortages of anesthesia [33]. No documented availability of ECT or rTMS in Djibouti or the Comoros.	In Sudan, awareness is moderate in urban areas, but professional training is limited [32]. ECT is used for severe conditions like schizophrenia and mania, but systematic evaluation is often lacking [33].

## Data Availability

Not applicable.

## Abbreviations

The following abbreviations are used in this manuscript:

DALYs	Disability-Adjusted Life Years
ECT	Electroconvulsive Therapy
GCC	Gulf Cooperation Council
MENA	Middle East and North Africa
rTMS	Repetitive Transcranial Magnetic Stimulation
tDCS	Transcranial Direct Current Stimulation
TRD	Treatment-Resistant Depression
UAE	United Arab Emirates
UK	United Kingdom
US	United States
WHO	World Health Organization
